# AfroColombian ethnicity, a paradoxical protective factor against Dengue

**Published:** 2016-09-30

**Authors:** Jorge Humberto Rojas Palacios, Alberto Alzate, Héctor Jairo Martínez Romero, Alberto Ignacio Concha-Eastman

**Affiliations:** 1 Grupo de Epidemiología y Salud Pública, Secretaría de Salud Pública Municipal de Cali, Alcaldía Municipal de Santiago de Cali, Cali Colombia; 2 Grupo de Investigación en modelos y métodos matemáticos para el control y vigilancia del dengue, Cali, Colombia; 3 Grupo de Investigación en Epidemiología y Servicios (GRIEPIS). Postgrado en Epidemiologia, Universidad Libre, Cali, Colombia; 4 Departamento de Matemáticas, Universidad del Valle. Cali, Colombia; 5 Alcaldía Municipal de Santiago de Cali, Cali, Colombia

**Keywords:** Ethnicity, ethnic groups, AfroColombian, African continental ancestry group, dengue, ecological, epidemiological surveillance, protective factors, risk factors

## Abstract

**Introduction:**

Dengue is a priority public health problem. During epidemics in Cuba and Haiti, ethnic African descendant population had lower risk of dengue, and the ethnic factor was proposed as a protective one.

**Objective:**

To determine the relation between the Dengue's cumulative incidence and the Afro-Colombian proportion in communities of Cali, during the epidemic of 2013.

**Methods:**

This study was conducted in Cali, Colombia. The design was ecological, using information from the National Census 2005 projected to 2013, from the National Administrative Department of Statistics (DANE), and the National Epidemiological Surveillance System. It was obtained the Pearson´s correlation coefficient between cumulative incidence and the proportion of Afro-Colombian population by communities. Additionally, the cumulative incidences of dengue were evaluated in two zones with different proportion of Afro-Colombian population. The association was also evaluated for aggregation bias, confounding by social variables, and interaction by area of ​​residence.

**Results:**

Dengue´s cumulative incidence was significantly lower for Afro-Colombians regardless of the proportion of Afro-Colombian population in the area of residence. The relative risk of dengue between non-Afro-Colombians and Afro-Colombians was 9.4 (95% CI=8.4-10.6) in zones with high proportion of Afro-Colombian population, while the relative risk of dengue was 4.0 (95% CI :3.6 - 4.4) in the zone with lower proportion of Afro-Colombian population. There was no evidence of aggregation bias or confounding in the association by social variables.

**Conclusions:**

The Afro-Colombian population had a significantly lower risk of getting dengue and its complications, compared with the non-Afro-Colombian population. The non-Afro-Colombian populations living in areas with a high proportion of Afro-Colombians increase their risk of dengue more than double, suggesting an asymptomatic viremic environment.

## Abstract (es)

**Introducción:**

el dengue es un problema prioritario en salud pública. Durante epidemias en Cuba y Haití, la población étnica afro-descendiente tuvo menor riesgo de dengue. Por ello, se propuso el factor étnico como protector.

**Objetivo:**

Determinar la relación entre la incidencia acumulada de dengue y la proporción de población Afrocolombiana de Cali, durante la epidemia de 2013.

**Métodos:**

Este estudio se realizó en Cali, Colombia. El diseño fue ecológico, con información del Censo Nacional 2005 y su proyección a 2013 del DANE y del Sistema de entre las incidencias acumuladas de dengue y la proporción de afrocolombianos, según comunidades. Adicionalmente, fueron evaluadas las incidencias acumuladas de dos zonas con diferente proporción de habitantes afrocolombianos. También se evaluó la presencia del sesgo de agregación, de confusión por variables sociales y de interacción según la zona de residencia.

**Resultados:**

Para afrocolombianos, la incidencia acumulada de dengue fue significativamente menor, independientemente de la proporción de población afrocolombiana en la zona de residencia. El riesgo relativo de dengue entre no-afrocolombianos y afrocolombianos fue 9.4 (IC 95%= 8.4-10.6) en la zona con más alta proporción de población afrocolombiana, mientras que el riesgo relativo fue 4.0 (IC 95%= 3.6-4.4) en la zona de más baja proporción de población afrocolombiana. No se evidenció sesgo de agregación ni confusión de la asociación por variables sociales.

**Conclusiones:**

La población afrocolombiana presentó un riesgo significativamente menor de enfermar por dengue y sus complicaciones en comparación con no-afrocolombianos. La población no-afrocolombiana que reside en zonas con alta proporción de población afrocolombiana aumenta el riesgo de dengue a más del doble, lo cual sugiere un entorno virémico asintomático.

## Introduction

 In Colombia, dengue is a priority public health problem. Twenty five million people living at altitudes of up to 2,200 m high above sea level are at risk, where there is a high infestation of the mosquito *Aedes aegypti *are at risk. In addition, four serotypes are circulating simultaneously since 1982. In the early 90's, cases rose from 5.2 cases/100,000 inhabitants to 18.1 cases/100,000 inhabitants. Lethality in the first decade of the century was approximately 1.17% 1; and in 2013, it reached 5.8 deaths per 100 cases of severe dengue 2.

 There are factors that increase the risk of developing severe dengue: the pre-existence of antibodies against a different serotype because of previous infection and the sequence of the infecting serotypes, being a woman 3; malnutrition: obese children with severe dengue have a worse prognosis, early age of infection: children have a risk that is 40 times higher than the risk in adults suffering severe dengue. As a protective factor 4,5, it has been observed that during epidemics of dengue, African-descendants had lower proportion of severe clinical forms and hospitalizations, even in conditions of equal access to health services. In Africa and Haiti, although different serotypes circulated simultaneously, there was no evidence of outbreaks in children 6,7. With these observations, it has been proposed the existence of a human gene that moderates the clinical expression of dengue infection among African-descendant individuals, but there have also been found differences in polymorphisms in the HLA Class I locus 8. These observations made in Cuba and Haiti 6,7,9  have not been corroborated elsewhere nor by other research groups as a contribution to the consistency of this association. Since the epidemic that occurred in Cali (Colombia) in 2010, it was described a low incidence of dengue in the AfroColombian population compared with the incidence in the rest of the population 10.

 This study aimed to determine differences in the dengue cumulative incidence among communities with different proportion of Afro-Colombians during the dengue epidemic in Cali, in 2013. 

## Materials and Methods

The study was conducted in Cali, Colombia, a city with a population of 2,319,684 people (98.4% in urban areas and 1.6% in rural areas) 11, which is located at 3°27'26" of latitude North and 76°31'42" of longitude West (Greenwich Meridian), at an altitude of 1,070 meters above the sea level, with an average temperature of 24.7° C, an annual precipitation of 1,019.2 mm 12; conditions that explain the persistence of *Aedes aegypti* and *A. albopictus* mosquitoes 13,14.

 Through an ecological study, the accumulated incidences of dengue were compared among communities categorized according to the proportion of the Afro-Colombian population taken from the Census of the National Administrative Department of Statistics (DANE for its name in Spanish) in 2005, and its projections for 2013 11. Furthermore, the distribution of population of the city in communes was taken from the Municipal Planning Office 13. The Dengue cases were obtained from the National Epidemiological Surveillance System (SIVIGILA) 15. 

The variable effect was the incidence of dengue, measured as the cumulative incidence of cases/10,000 inhabitants; and the variable exposure was ethnicity, measured as a percentage of Afro-Colombians and non-Afro-Colombians in the communities of the city of Cali, according to the DANE definitions. The lethality rate corresponds to the number of dengue deaths occurred per 100 cases of severe dengue. 

### Data source 

 The database of dengue and severe dengue provided by the SIVIGILA of Colombia was reviewed in 2013 15. By Decree 3518 of October 9^th^, 2016, the Ministry of Social Protection established and regulated the SIVIGILA 16, in order to guide policies and planning in public health, making decisions for the prevention and control of diseases and health risk factors, optimizing the monitoring and evaluation of interventions, rationalizing and optimizing available resources and ensuring the effectiveness of actions in this area, moving towards the protection of individual and collective health.

###  Colombian Public Health Surveillance System

 The Public Health Surveillance System has an essential function associated with the state and civic responsibility for health protection, consisting of systematic and continuous process of collection, analysis, interpretation and dissemination of specific data related to health as the events of Interest in Public Health, for use in the planning, implementation and evaluation of public health practice.

Events of Interest in Public Health are those events considered important or transcendent for public health by the Ministry of Health and Social Protection in Colombia, taking into account criteria of frequency, severity, epidemiological behaviour, prevention possibilities, cost-effectiveness of interventions, and public interest; they also need to be controlled with public health measures.

 The implementation and development of the Public Health Surveillance System are under the responsibility of the Ministry of Health and Social Protection, the National Institutes of Health (INS), Surveillance Food and Drug Administration (INVIMA), Departmental, District and Municipal Departments of Health, that exercise functions of supervision and sanitary control to ensure the protection of public health and compliance with the provisions according to established standards; furthermore, advancing the procedures and applying the sanctions as need. Also, Administrative Entities of Health Benefit Plans, Reporting Units and Generating Primary Data Units are elements of this health surveillance structure.

 The Primary Data Generating Units (UPGD) are public or private entities that capture the occurrence of events of interest in public health and generate useful and necessary information for the purposes of the SIVIGILA. These Units are responsible for the mandatory weekly notification, the timely, continuous, accuracy and quality of the information and reports that SIVIGILA requires. Failure to comply with these provisions will result in disciplinary and other administrative sanctions in accordance with legal regulations.

 The Notifying Unit is responsible for the investigation, confirmation and configuration of events of interest in public health, based on information provided by the Primary Generating Data Units and any other information obtained through epidemiological methods. 

**Surveillance'stargets**. The information obtained as a result of the implementation of the Health Surveillance System is used to fulfil the following purposes: a) to estimate the magnitude of the events of interest in public health; b) to detect changes in patterns of occurrence, distribution and propagation of events under surveillance in public health; c) to detect outbreaks and epidemics and target specific control actions; d) to identify risk factors and protective factors related to the events of interest in health and population groups exposed to these factors; e) identify needs of epidemiological research; f) to provide health planning and defining prevention and control measures; g) to facilitate monitoring and evaluation of health interventions; h) to guide actions to improve the quality of health services; and i) to guide policy-making in public health..

 Information flows between the actors in the system of public health surveillance in Colombia, rising from the Municipal local level with 5,500 generating primary data units UPGD of which 157 are in Cali and, 1,122 municipal notifying units at the departmental level; 36 departmental units reporting to the INS. There, the data debugging is made as well as analysis, reports, monitoring, assessing of information, definition of strategies and standardizing of monitoring techniques that supports the management, and feedback of the system. . 

The Ministry of Health and Social Protection as the highest authority leads, coordinates, regulates and examines the epidemiology situation. This feature in the information flow assigns each of the actors particular functions defined in the surveillance system and national public health regarding the use of data and operation of the application.

 Given that the National Surveillance System is based on the individualized report of new cases of a certain event of interest in public health, it was necessary to have technological tools that streamline and simplify this procedure for the SIVIGILA 2016 application system, which for clarity and understanding is accompanied by the corresponding operation manual 17.

### Inclusion criteria

This study included every person that went to health institution and a physician found fever ≥38.2 ^o^C and two or more of the next symptoms or signs: headache, retro-orbital pain, myalgia, arthralgia (fever break-bone), rash maculopapular, after discarding surgical pathologies , focal infection, malaria, leptospirosis and influenza; furthermore the laboratory confirmed dengue antibodies DENV IgM by ELISA positive after five days of staying the fever, NS1 antigen positive by ELISA or DENV-1-4 positive by RT-PCR assay in the first five days of starting the fever. Also, were included cases by epidemiological link, defined as those that occurred at a distance of up to 200 meters of a laboratory-confirmed case within 3 weeks (21 days) before or after the confirmed case, and in accordance with the national guidance for care of patients with dengue and the protocol of Public Health Surveillance 1,13. Fatal cases were obtained from SIVIGILA, death certificates from the vital statistics area, and the records of mortality analysis of every probable death case of Dengue.

### Biases 

The classification of ethnicity could introduce a classification's bias, when the physicians decide to classify the case only on the basis of phenotypic characteristics. But, in our study, ethnicity was classified according with the self-recognition of each patient. 

**Aggregation bias.** Attributable in ecological studies to the fact that the association found at added level does not necessarily correspond to an association at individual level, so it must be assessed.

### Analysis plan

 The data base was adjusted according to the algorithm of the National Institute of Health of Colombia (INS) to identify and eliminate duplicated cases, those discarded by laboratory, and those from out of town 18. Dengue cases were obtained by communities, age and ethnicity: Afro-Colombians and non-Afro-Colombians.

 The populations were obtained as follows: the difference between the total population and the registered total of dengue cases resulted in the total of non-cases; the difference between the total for Afro-Colombians according to DANE and the Afro-Colombian cases according to SIVIGILA records resulted in the Afro-Colombian non-cases; and the difference between the total of non-Afro-Colombians according to DANE and the non- Afro-Colombian cases according to SIVIGILA resulted in the non-Afro-Colombian non-cases. 

 The proportion of Afro-Colombians in the communities was calculated. The communities were classified into two living areas in proportion to the Afro-Colombian population (communities with high proportion of Afro-Colombians containing 30% or more and communities with low proportion of Afro-Colombians containing less than 30%). 

 The cumulative incidence of dengue in the communities and the two Afro-Colombian zones (high and low proportion) was adjusted by population and ethnic group using the direct method, because the population pyramids and the proportion of Afro-Colombians contained in the communities are different. The reference population was that of the city of Cali by age groups (Table 1).

**Table 1.** Population distribution absolute and relative according to ethnic and age groups*. Cali, 2013.

	Cali		Afro-Colombians		Non-Afro-Colombians	
Age range	n	%	n	%	n	%
<15	542,573	23.4	148,833	24.5	393,740	23.0
15-44	1,104,585	47.6	296,276	48.7	808,309	47.2
45-64	491,419	21.2	111,749	18.4	379,670	22.2
≥65	181,107	7.8	50,897	8.4	130,210	7.6
Total	2,319,684		607,755		1,711,929	

*DANE. Projections according to the 2005 census

 It was evaluated the level of relation of the proportion of Afro-Colombian population with the cumulative incidence of dengue among the communities, through the correlation method, and the value of the Pearson's statistical coefficient r was obtained.

 Next, the cumulative incidence of dengue for Afro-Colombians and non-Afro-Colombians was calculated and the first was taken as the reference category because it was the lowest incidence. That was compared with the cumulative incidence of dengue in the non-Afro-Colombian category, with the cumulative incidence in the "zone with low proportion of Afro-Colombians" category and finally with the cumulative incidence of dengue in the "zone with a high proportion of Afro-Colombians" category. The relative and the attributable risks were estimated with their respective confidence intervals. 

**Figure 1.**
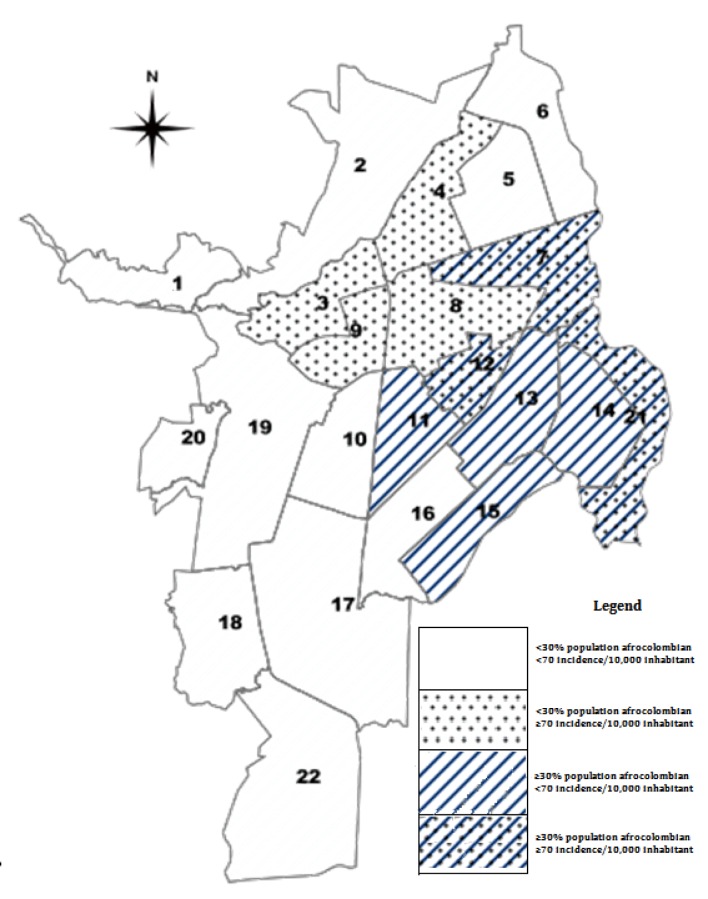
Overlay between distribution of population Afro-Colombians and dengue risk by communes, Cali, 2013. Four groups of communes were grouped according to AfroColombian population distribution and the risk of dengue.

 Two maps were compared: One, with a distribution of communities according to the proportion of Afro-Colombians contained in them; and another map, with the distribution of dengue risk in the communities. The maps were constructed using the GeoDa version 2012, Software of geospatial analysis 19 and posteriorly overlaid in one (Fig. 1).

 An assessment for aggregation bias was made by evaluating the consistency of the association. The proportion of Afro-Colombians was compared between the group cases and non-cases. The association was assessed for potential confusion caused by the following variables: area of residence according to the proportion of Afro-Colombians, population density, density of (land) lots, density of schools, access to safe drinking water, cleaning services and, building permits. Each one was stratified in three categories. This information was available in the Administrative Department of City Planning of Cali. It was evaluated for interaction by comparing the homogeneity of ethnicity effects on the incidence of dengue in each area, and also by the method of comparing the observed and expected joint effects of ethnicity on the incidence of dengue. The statistical analysis was performed using the Stata program 6. 

##  Results 

### Participants and descriptive data

 In Cali, 26.2% of the population is self-proclaimed Afro-Colombian, equivalent to 607,755 people out of 2,319,684 inhabitants of Cali (Table 1). C7, C11, C12, C13, C14, C15 and C21 are communes located to the East of the city. These communes contained populations with a ratio of Afro-Colombians above 30%. This zone with high proportion had 41.7%; while the rest of the communes of the city contained proportion of Afro-Colombians below 30%. This other zone with low proportion had 16.5% (Fig. 1).

**Figure 2.**
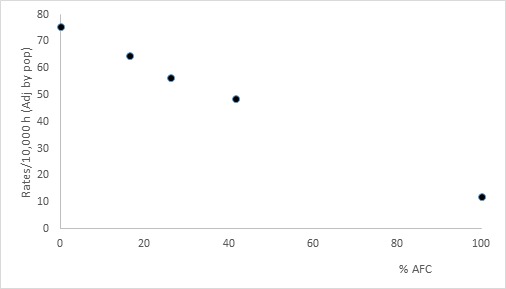
Dengue cumulative incidence according to % populations AfroColombian. Cali, 2013. Pearson correlation was -0.99 (CI 95%= -0.98 - -1.0). Dots indicated the dengue cumulative incidence by levels of proportions of populations of Afro-Colombians; left dot is the dengue incidence for only Non-Afro-Colombians, next dot is the dengue incidence for low proportions of populations of Afro-Colombians; central dot is the dengue incidence for Cali population; next dot is the dengue cumulative incidence for high proportion of Afro-Colombians; right dot is the dengue cumulative incidence for only population Afro-Colombians.

During 2013, there was an epidemic with 13,433 cases of dengue 20. The cumulative incidence of dengue in the general population of Cali was 56.7/10,000 inhabitants (CI 95%= 55.7-57.6). When cumulative incidence was adjusted by ethnicity, it was estimated at 57.6/10,000 inhabitants (CI 95%= 44.1-71.1). The cumulative incidence of only Afro-Colombians was 11.5/100,000 inhabitants (CI 95%= 10.7-12.4/10,000 inhabitants), the cumulative incidence of only non-Afro-Colombians was 70.4/100,000 inhabitants (CI 95%= 69.2-71.7/10,000 inhabitants). The cumulative incidence in the zone with high proportion of Afro-Colombians was 48.6/10.000 inhabitants (CI 95%= 47.7-49.5/10,000 inhabitants) and the cumulative incidence in the zone with low proportion of Afro-Colombians was 64.7/10,000 inhabitants (CI 95%= 63.7-65.8/10.000 inhabitants). Two maps were constructed on the concentration of Afro-Colombians and the risk of disease and then, they were overlaid. A pattern of opposite or inverse distribution between the concentration of Afro-Colombians and the risk of dengue disease can be observed; so, the communities with the higher proportion of Afro-Colombian population showed the lowest risk of dengue (Fig. 1). This result was verified with the negative and perfect Pearson´s correlation coefficient r value equal to -0.99 (CI 95%= -0.98--1.0) (Fig. 2).

### Ecological association dengue-population

 We evaluated the ecological association between cumulated dengue incidence and ethnicity comparing the proportions of Afro-Colombian population between cases and non-cases of dengue and so, discarding ecological fallacy. We found that these proportions of Afro-Colombians were different. It was found that the proportion of Afro-Colombians among cases was 5.5%, while in the non-cases it was higher (26.2%) (Table 2). This means that among the cases, there are proportionally fewer Afro-Colombians than among the non-cases; and that they do not have the same probability for a dengue case to appear. It was preserved the same association and direction that those found in the ecological study, indicating that there was no ecological fallacy; therefore, the association found at the ecological level can be inferred at individual level.

**Table 2.** Evaluation of ecological fallacy through of the comparison of the proportion of AfroColombian population between cases and non cases.

Disease	Dengue			Non dengue		
	n	%	CI 95%	n	%	CI 95%
AfroColombian	699	5.5	5.1-5.9	605,146	26.2	26.2 -26.3
Non AfroColombian	12,066	94.5	94.1-94.9	1,701,773	77.8	73.7-73.8
Total	12,765	100.0		2,306,919	100.0	

This means that among the cases, there are proportionally fewer Afro-Colombians than among the non-cases; and that they do not have the same probability that a dengue case appears. It was preserved the same association and direction that those found in the ecological study, indicating that there was no ecological fallacy; therefore the association found at the ecological level can be inferred at individual level.

The crude and age-adjusted cumulative incidence were higher and significant statistically in the zone with high proportion of Afro-Colombians. However, when adjusted for ethnicity, this difference statistical disappeared (Table 3).

**Table 3.** Dengue cumulative incidence adjusted by age and ethnic group according to Afro-Colombians zones. Cali, 2013.

Zone	Cumulative incidence*					
	Crude	95% CI	**Age	95% CI	*** Ethnic group	95% CI
Zone with high Afro-Colombians	64.1	62.8-65.4	64.7	63.7-65.8	111.8	99.0-124.6
Zone with low Afro-Colombians	49.4	47.9-50.9	48.6	47.7-49.5	124.1	111.3-136.9
Attributable Risk	16.1	12.8-16.7	16.2	14.8-17.5	12.3	5.8-149.2
Relative Risk	1.3	1.25-1.35	1.3	1.3-1.4	1.1	0.98-2.67

*by 100,000 inhabitants; **Adjust by age; ***Adjust by age and ethnic group. Adjusts were made by direct methods using the Cali population like as reference

### Evaluation of confusion 

The direction and strength of the association between dengue incidence and ethnicity remained when evaluating different strata of possible confounding variables. It was observed heterogeneity of dengue incidence in the following strata: population density and density of (land) lots per communities, suggesting a synergy and thereby effect amplification (Table 4).

**Table 4.** Evaluation of possible confusers of the association between ethnic group and cumulative incidence dengue. Cali. 2013.

Evaluated determinant for confusion	Strata	Non-Afro-Colombians	Afro-Colombians	Attributable risk	Relative risk	95% CI
Cali		70.4 (69.2-71.7)	11.5 (10.7-12.4)	58.9	6.1	5.7-6.6
Population Density (Inhabitant/Hc)	9.7-99.6	67.51	20.04	47.5	3.4	2.9-3.9
	193.2-228.8	72.05	15.58	56.5	4.6	4.1-5.2
	279.9-333.3	72.95	7.45	65.5	9.8	8.6-11.1
Land Lots density (Average Land Lot/commune)	670	88.01	15.72	72.3	5.6	4.8-6.5
	1,236	74.52	12.80	61.7	5.8	5.2-6.5
	5,230	66.38	10.32	56.1	6.4	5.8-7.2
School Density (Average School/commune)	101.1	64.90	10.65	54.3	6.1	5.4-6.9
	129.45	67.43	9.42	58.0	7.2	6.3-8.1
	161.90	84.28	15.04	69.2	5.6	4.9-6.4
Access to safe water (Subscribers Average/commune)	11,921	80.70	18.37	62.3	4.4	3.7-5.2
	231,982	83.21	10.34	72.9	8.0	7.1-9.1
	371,561	59.82	10.59	49.2	5.6	5.0-6.4
Solid waste collection services (Subscribers Average/commune)	13,145	80.90	14.09	66.8	5.7	4.8-6.9
	25,489	83.11	11.44	71.7	7.3	6.4-8.2
	40,690	59.82	10.59	49.2	5.6	5.0-6.4
Building Licenses (Average Building Licenses/commune)	23	76.10	13.06	63.0	5.8	5.0-6.7
	48	71.08	7.46	63.6	9.5	8.2-11.0
	95	68.42	15.89	52.5	4.3	3.8-4.8

### Identification of interaction or effect modification

 The stratification of the city into two zones: One, the zone with a high proportion of Afro-Colombians (zone A) and two, the zone with a low proportion of Afro-Colombians (zone B) and their analysis determined that Afro-Colombians always had lower cumulative incidence of dengue than non-Afro-Colombians (Table 5). Furthermore, it was observed a significant heterogeneity in the strength of the association between ethnicity and the incidence of dengue in the two zones evaluated using the strategy of assessment of homogeneity of effects in zone A (RR= 9.4) and zone B (RR= 4.0) as well as comparing the observed and expected joint effects (additive model: expected effect: 94.4); observed effect: 59.2; multiplicative model: expected effect 19.3; observed effect 8.2 (Table 6).

**Table 5.** Accumulated Incidence of Dengue according to ethnic group and residence zone.

	Ethnic group							
	Afro-Colombians				Non-Afro-Colombians			
Zone	Population	Cases	Cumulative incidence*	95% CI	Population	Cases	Cumulative incidence*	95% CI
Afro-Colombians (high proportion)	372,249	306	8.2	7.3-9.1	504,305	3,911	77.6	75.1-80.1
Afro-Colombians (low proportion)	233,596	393	16.8	15.2-18.5	1,209,533	8,155	67.4	66.0-68.9
Total	605,845	699	11.5	10.7-12.4	1,713,839	12,066	70.4	69.2-71.7

Pop= Population

*adjusted/10,000 inhabitant

**Table 6.** Evaluation of interaction between ethnic group and zones with high and low proportion Afro-Colombians.

	Homogeneity					
	Additive Model		Multiplicative Model			
Zones	Attributable Risk	CI 95%	Relative Risk	CI 95%		
Afro-Colombians (high proportion)	69.3	66.7-71.9	9.4	8.4-10.6		
Afro-Colombians (low proportion)	50.6	48.4-61.8	4.0	3.6 - 4.4		
Comparison of the joint expected and observed effects						
	Incidence/10,000 inhabitants		Attributable Risk		Relative Risk	
Zones	Afro-Colombians	Non-Afro-Colombians	Afro-Colombians	Non-Afro-Colombians	Afro-Colombians	Non-Afro-Colombians
Afro-Colombians (low proportion)	16.8(15.2-18.5)	67.4(66.0-68.9)	0 (-1.2-1.2)	50.6 (49.5-51.7)	1.0 (0.9-1.2)	4.0 (3.6-4.4)
Afro-Colombians (high proportion)	8.2(7.3-9.1)	77.6(75.1-80.1)	-8.6(-7.6-9.6)	60.8(59.2-62.2)	0.5(0.4-0.6)	4.7(4.2-5.1)
Joint expected effects			42.0 (40.5-43.5)		2.0(1.6-2.4)

**Figura 3.**
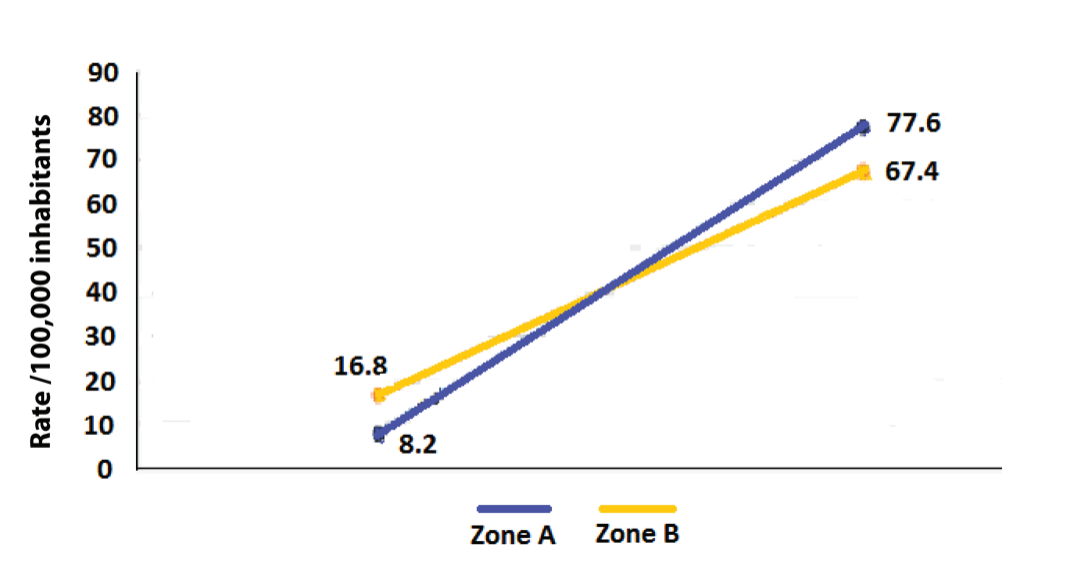
Interaction or effect modification of ethnic over dengue incidence by the proportion of population AfroColombian in communities.

The confidence intervals indicated that the differences were significant statistically and that the modification of effect exist (Fig. 3, Table 6). 

 For non-Afro-Colombian people that resided in the zone with higher proportion of Afro-Colombians, the dengue risk was increased nearly double than for those who lived in low proportion Afro-Colombian zones (synergy). This effect modification of ethnicity on the incidence of dengue by area of ​​residence was of additive and multiplicative type, for both, the method of the homogeneity of the effects and for the method of comparing the observed and the expected joint effects (Table 6).

 Of 431 cases of severe dengue, 402 occurred among non-Afro-Colombians (85.7) and 29 (14.3%) in Afro-Colombian. Twelve deaths occurred; one (8.3%) in Afro-Colombians and 11 (91.7%) in non-Afro-Colombians. Lethality for severe dengue was 2.7% in Afro-Colombian and 3.4% in non-Afro-Colombians. These differences in fatality rates were not statistically significant (OR= 0.8; CI 95%= 0.1-6.0).

## Discussion

 The risk of dengue for an inhabitant of Cali is similar in either of the two studied zones with different proportions of Afro-Colombians when it is adjusted by ethnicity. But, the ecological study, with the analysis of population and individual risk showed that there is an association between ethnicity and dengue. The Afro-Colombians had a significantly lower risk of dengue disease than the non-Afro-Colombians. 

The present study found a gradient in the incidence of dengue in proportion to Afro-Colombians in communities and living areas (Fig. 1). In Cali, Afro-Colombians have a concentrated distribution in the East and Center-East parts of the city. It was also found a negative interaction of the effect of ethnicity on the incidence of clinically apparent dengue when it was stratified in the two living zones (high and low proportion of Afro-Colombian population). Non-Afro-Colombians in Cali had a higher incidence of dengue in comparison with Afro-Colombians and this difference increased when non-Afro-Colombians live in an area of ​​high Afro-Colombian proportion. This shows that the dengue virus also circulates in the Afro-Colombian population. The difference in incidence found could be explained because that Afro-Colombian population showed no recognizable symptoms, even though it was infected; therefore, this population did not consult nor they were registered as dengue cases in the clinical and epidemiological statistics of SIVIGILA. These findings agree with the results found in Cuba and Haití 7,8.

 The lethality for severe dengue in Cali, in 2013 20 was 2.8%, lower than the national average (5.8%) 2, but higher than the internationally established standard (2%) 12, Confirmed deaths from severe dengue, occurred only in one Afro-Colombian patient who had sickle cell anemia as a risk factor. It was not found a statistically significant difference in lethality by ethnicity (RR= 0.8; CI 95%= 0.1-6.0) 20. Lethality in dengue is directly related to failures in opportunity for consultation by the patient, timeliness and quality of care for patients with dengue to prevent progression to severe dengue 1. The membership coverage in the health insurance system, in Cali, in 2013, was 93.2%; and the non-members are covered by a state network of social entities that form the Public Health System. This shows that access to health services and quality of health care do not explain the differences in the incidence of dengue by ethnicity.

Studies in Cuba have found association between ethnicity and the morbidity and mortality caused by dengue and severe dengue 5,6,9; which has promoted the recognition of ethnicity as a possible risk factor for the development of severe dengue 7,8.

In Haiti, this same phenomenon of the relationship between ethnicity and dengue was documented 7. Non-Afro-descendants showed a more vigorous cellular immune response specific for dengue virus antigens. These epidemiological and laboratory observations are of significant interest and they match the low reporting of dengue in Afro-descendants in the Caribbean.

 Considering the central role of immunological mechanisms in the pathogenesis of the disease, genes associated with the immune response, such as the HLA complex should be considered. Significant differences in some HLA antigens between Afro-descendants and non-Afro-descendants have been found, which possibly are involved in the low-risk observed in Afro-Colombians 8. Infectious diseases can act as a strong selective influence in shaping human evolution and the genetic structure of populations 9.

 Very similar proportions of positivity for antibodies against dengue between Afro-Colombians and non-Afro-Colombians in the Antioquia's Uraba, region of Colombia have been found 21, which points to equal rates of virus infection. There were no statistically significant differences between Afro-Colombians and white people (OR= 1.29; CI 95%= 0.91-1.82); or Indians and white people (OR= 1.82; CI 95%= 0.62-5.28). If the Afro-Colombians of Cali share the same genetic pool of the Afro-Colombians of Uraba, it reinforces the hypothesis of genetic resistance of Afro-Colombians in Cali to severe dengue and dengue, because they do not manifest any signs or symptoms symptoms, which would explain the distribution of dengue, severe dengue and confirmed dengue deaths according to the characteristics of the population and its geographical distribution in Cali.

 The results of this study in Cali will contribute to readjust public health programs and medical care services to planning and the control of dengue's epidemics; so, improving the patient´s attention, prevention, orientation of control actions; epidemiological, entomological, virological and serological surveillance, and its impact on planning of vaccination strategies. The epidemics occurred in Cali 2010 and 2013 were characterized by the suddenness of their occurrence, with a rapid increase of cases within a few weeks 10,20. What it could have happened is that dengue was unnoticed among the Afro-Colombians in the first stage of the epidemic with a high number of infected people, which thereby would increase mosquito's infection locally. The epidemic propagated and it was only epidemiological and clinically evident much later, when enough of non Afro-Colombians consulted the health services and was reported to the surveillance system. Social mobility, for reasons of labor, education or business, would increase the infection of mosquitoes and indirectly of human populations from predominantly Afro-Colombian zones toward predominantly non-Afro-Colombian zones. That would explain the explosive multicenter character of the epidemic in the city 10,20.

The association between Dengue and ethnicity is important in countries like Colombia 22, which have regions with a high proportion of Afro-Colombians, because if they are asymptomatic in high proportion, the viral circulation will become obvious only in advanced stages of the epidemic, when it affects a large proportion of non-Afro-Colombians, who does show symptoms.

In Nicaragua 23, it was found that the asymptomatic / symptomatic ratio ranged from 3-16, but they did not report the ethnicity distribution. Applying this expansion factor and the asymptomatic-symptomatic ratio, in Cali almost all of the population would have been affected.

Although the ecological association may exactly reflect a causal link between a risk factor and an effect, the ecological fallacy may limit its usefulness. The ecological fallacy is a bias that may occur due to the association between variables at the aggregate level, and it does not necessarily represent the association at an individual level 24. In studies of factors that explain the transmission of certain infectious diseases for which herd immunity is important, the study of individuals as units of observation (case-control, cohort, etc.) may be inappropriate. Ecological studies may be the only way to study these patterns and the risk factors for transmissibility.

### Limitations of the study

 The reported cases in this study are based on a National Public Health Reporting Surveillance System, where it is recognized that not all the cases are captured, because there are patients who do not attend medical consultation because they were classified as viruses or fever of viral aetiology. However, the monitoring system in Cali guarantees continuity, training of health professionals on the new protocol of epidemiological surveillance for dengue that started in 2009, in continuous training on the filling of formats and adherence to guidelines for the assistance of the cases. This, added to the dramatic clinical picture, which is popularly called fever bankruptcy-bones, to the diffusion through mass media about the danger signs and the risk of advancing to severe forms and death if it is not diagnosed timely and if an adequate hydration is not given to the patient, it gives us confidence that during the epidemic that we investigated, the majority of cases were recorded.

## Conclusions

The Afro-Colombians has a risk to manifest clinical symptoms of dengue that is 6.1 times lower than that for non-Afro-Colombians people (Table 5). It was evidenced a modification of the effect of ethnicity on the incidence of the disease in the area of ​​residence according to the proportion of Afro-Colombians. While being Afro-Colombian protects the individual against dengue and its severe forms, paradoxically their non-Afro-Colombians neighbours have an even greater risk of getting the disease if they live with neighbours of the same ethnicity.

The consistency of the association between ethnicity and dengue has been not documented by other authors with this methodology in other parts of the world; a biological gradient was observed in the incidence of the disease with the proportion of Afro-Colombians, and it was found biological plausibility according to the existing basic knowledge.

 This difference in incidence of dengue between Afro-Colombian and non Afro-Colombian populations because of differences in the clinical manifestations should be taken into account when comparing the incidence or prevalence of dengue disease among neighbourhoods, communities, cities, countries or regions during epidemics. Also, when the studies impact on medical-health interventions and vaccination have been evaluated; because not only they should make adjustments to these cumulative incidences by distributing their age groups, but also they should take into account the proportion of Afro-Colombians contained in the population. For infection studies, the results should be based on serological data.

 This study will allow a new approach to the study of outbreaks of dengue; prevention; targeted and prioritized control actions; epidemiological, entomological, virological and surveillance; on medical care; on the impact assessment and the planning of vaccination strategies.

In this study, the ecological correlation between the cumulative incidence of dengue areas and the proportion of Afro-Colombians was negative and perfect. The same association was found at the individual level, by which it was concluded that there is no ecological fallacy, and that the ecological association can be inferred at individual level. 
